# Extended Cognition and the Internet: A Review of Current Issues and Controversies

**DOI:** 10.1007/s13347-016-0250-2

**Published:** 2017-01-14

**Authors:** Paul Smart

**Affiliations:** Electronics and Computer Science, University of Southampton, Highfield, Southampton, SO17 1BJ UK

**Keywords:** Internet, World wide web, Extended cognition, Extended mind, Epistemology, Cognitive science

## Abstract

The Internet is an important focus of attention for those concerned with issues of extended cognition. In particular, the application of active externalist theorizing to the Internet gives rise to the notion of Internet-extended cognition: the idea that the Internet can (on occasion) form part of an integrated nexus of material elements that serves as the realization base for human mental states and processes. The current review attempts to survey a range of issues and controversies that arise in respect of the notion of Internet-extended cognition. These include the issue of whether the Internet, as a technological system, is able to support real-world cases of cognitive extension. It also includes issues concerning the cognitive and epistemic impacts of the Internet. Finally, the review highlights a range of issues and concerns that have not been the focus of previous philosophical attention. These include issues of ‘network-extended cognitive bloat’, ‘conjoined minds’, and an entirely new form of cognitive extension that goes under the heading of ‘human-extended machine cognition’. Together, these issues serve to highlight the value and importance of Internet-extended cognition to contemporary philosophical debates about the extended mind. In particular, the notion of Internet-extended cognition has the potential to highlight points of philosophical progress that are not easily revealed by the kind of technologically low-grade cases that tend to animate the majority of philosophical discussions in this area.

## Introduction

A common focus of discussion in the sciences of the Web and Internet is the issue of ‘boundary dissolution’. The Internet is thus sometimes seen to be blurring the traditional distinction between ‘work’ and ‘leisure’ (Gant and Kiesler 2001), between the ‘public’ and the ‘private’ (Thompson 2011), between the ‘real’ and the ‘virtual’ (Floridi 2014), between the ‘human’ and the ‘machine’ (Lupton 2013; Warwick 2015; Verbeek 2009), and between the ‘social’ and the ‘technological’ (see Smart and Shadbolt 2014). An additional form of boundary dissolution can be found in debates regarding the cognitive and epistemic impacts of the Internet. The main focus of attention, in this case, is the extent to which the Internet supports a form of cognitively potent bio-technological hybridization, one in which the informational and technological elements of the Internet can be seen as literal elements of the physical fabric that realizes human mental states and processes. The philosophical backdrop to this debate centers on the notions of active externalism, extended cognition, cognitive extension and the extended mind (Clark and Chalmers 1998; Clark 2008; Menary 2010).^1^ What all these locutions have in common is a commitment to the idea that the causally active physical vehicles of cognitive states and processes can (on occasion) extend beyond the biological boundaries of the individual cognitive agent to include elements drawn from the surrounding technological and social environment. When we apply this idea to the Internet, we arrive at the notion of ‘Internet-extended cognition’, or the idea that the Internet can (on occasion) form part of an integrated nexus of material elements that participates in the mechanistic realization of human mental states and processes (see Smart 2012).

Extended cognition has emerged as a major focus of theoretical and empirical interest for those working in the sciences of the mind. One of the reasons for this is its ability to account for the rather distinctive set of cognitive capabilities that are deemed to be the hallmark of human cognizing. Cognitive extension is thus seen to lie at the root of an important form of cognitive enhancement for our species (Clark 2008), and this makes the Internet a critical focus of attention for those who are concerned with issues of cognitive performance and cognitive well-being (e.g. Loh and Kanai 2016;Carr 2010;Wegner and Ward 2013;Smart 2013). Inasmuch as we accept the idea that the Internet forms part of the supervenience base for human mental states and process, then it is clearly important that we seek to understand the way in which the Internet is poised to help (or hinder) our species-specific cognitive capabilities.

Issues of Internet-extended cognition are also relevant to philosophical debates regarding the foundational notion of cognitive extension. In particular, the Internet has the potential to reveal issues and concerns that might not otherwise have been brought into the philosophical spotlight. Such issues and concerns are of crucial importance inasmuch as they help to shape the course of future philosophical debates.

The present paper attempts to survey at least some of the issues and controversies that surround recent attempts to apply active externalist theorizing to the Internet. For the most part, the focus of the review is restricted to issues that have arisen as the result of previous philosophical work (Staley 2014; Halpin 2013; Halpin et al. 2010; Smart 2012; 2014; in press; Smart et al. in press). Beyond this, however, the review also highlights issues that are (at best) only weakly represented in the current philosophical literature. These include issues of ‘network-extended cognitive bloat’ (see Section 3), ‘conjoined minds’ (see Section 4) and a new form of extended cognition that goes under the heading of ‘human-extended machine cognition’ (see Section 7).

## Web-Extended Minds

Although there have been a number of attempts to analyze the Internet from an active externalist perspective (see Staley 2014;Smart et al. in press), by far the greatest level of attention has been directed to a specific application of the Internet, namely the World Wide Web (Smart 2012; 2013; 2014; Halpin et al. 2010; Halpin 2013). Such work is relevant to the present discussion in the sense that the Web is commonly seen as a component of the Internet. The issues that arise in relation to Web-based forms of cognitive extension are thus part of a broader array of issues that are relevant to the more general topic of Internet-extended cognition.

The most explicit application of active externalist theorizing to the Web comes in the form of the ‘Web-extended mind hypothesis’ (Smart 2012). This is the idea that ‘the technological and informational elements of the Web can (at least sometimes) serve as part of the mechanistic substrate that realizes human mental states and processes’ (Smart 2012, p. 447). Although on the surface this looks to be a rather straightforward application of active externalist theorizing to a particular kind of technological system, the term ‘Web-extended mind’ is one that warrants a degree of careful analytic scrutiny. In particular, previous treatments of Web-extended cognition have tended to overlook the need to provide a clear and explicit definition of what is meant by the ‘Web’. This is important, because in the absence of an understanding of what the Web is, it is difficult, to say the least, to make sense of the term ‘Web-extended mind’. Part of my aim in this section is to clarify our understanding of what is meant by the terms ‘World Wide Web’, ‘Web-based system’, and ‘Web-extended mind’. In particular, I suggest that we should see a Web-extended mind as a bio-technologically hybrid cognitive organization that leads a dual life as *both* a Web-based system and an extended cognitive system (see Section 2.1).

Another aim of this section is to explore the extent to which the Web is able to support real-world cases of cognitive extension. Does the nature of our interaction with the Web allow for the emergence of actual extended cognitive systems, or are Web-extended minds merely a matter of theoretical interest and speculation? One way of making progress on this issue is to refer to the criteria that have been used to evaluate putative cases of cognitive extension. Perhaps the most well known of these criteria (although by no means the only ones^2^) are those proposed by Clark and Chalmers (1998) as part of their seminal work on the extended mind. The criteria in question are informed by a classic thought experiment involving a mnemonically impaired individual, called Otto, and a conventional, paper-based notebook. As is noted by Clark (2011), the aim of this particular thought experiment was to

...convince the reader that, under certain conditions, the coarse functional role of a bio-external encoding could be sufficiently similar to that of a persisting internal encoding as to mandate similar treatment, revealing the non-biological resource as part of the physical machinery underpinning some of an agent’s genuine mental states. (Clark 2011, p. 448)

As Clark and Chalmers are quick to note, however, not every form of bio-technological or bio-artifactual coupling seems to invite treatment in cognitive terms. ‘There would’, as Clark (1997) suggests, ‘be little value in an analysis that credited me with knowing all the facts in the *Encyclopaedia Britannica* just because I paid the monthly installments and found space for it in my garage’ (p. 217).

What, then, are the conditions under which we should count a set of bio-external resources (such as Web resources) as proper parts of the machinery of the mind? In answering this question, Clark and Chalmers (1998) proposed a set of criteria that have come to be known as the ‘trust and glue’ criteria (see Clark 2010b). The criteria, as presented by Clark (2010b, p. 46), are as follows:

- ‘That the resource be reliably available and typically invoked’. **[Availability Criterion]**
- ‘That any information thus retrieved [from the resource] be more or less automatically endorsed. It should not usually be subject to critical scrutiny (unlike the opinions of other people, for example). It should be deemed about as trustworthy as something retrieved clearly from biological memory’. **[Trust Criterion]**
- ‘That information contained in the resource should be easily accessible as and when required’. **[Accessibility Criterion]**

One way of evaluating the extent to which the Web is able to support extended cognition is thus by reflecting on the kinds of interaction that we currently have with the Web. We can then assess whether these kinds of interactive engagement satisfy the demands of the trust and glue criteria. This is the approach adopted by the current section. Although there is no doubt much more work to be done here—in particular, there are a number of other criteria that could be the focus of future analytic efforts (see Heersmink 2015; Clowes 2015; Sterelny 2010; Palermos 2014b)—this kind of analysis does reveal a range of issues that are likely to shape the profile of future philosophical debates in this area.

### Cognitive Systems and the Web

In order to understand what is meant by the notion of a Web-extended mind, we first need to understand what is meant by the ‘Web’. In a general sense, the Web can be defined as follows:

(**World Wide Web**) The Web is a set of globally-distributed resources, where the resources in question are identified using Uniform Resource Identifiers (URIs) and accessed using a specific Internet protocol, namely HyperText Transfer Protocol (HTTP).

Note that nothing in this definition constrains the nature of the resources that comprise the Web: anything that is accessed using a combination of URIs and HTTP is, by definition, a Web resource and thus forms part of what we call the Web. Having said that, our conventional use of the Web centers on a particular set of resources, namely hypertext files (or Web pages). These ‘documents’ rely on a specific kind of markup language, such as HyperText Markup Language (HTML), and they often include references to other (embedded) resources, such as Cascading Style Sheet (CSS) specifications, JavaScript code and multimedia content (e.g. images and video). The Web can, however, consist of other kinds of resources. Thus, in addition to seeing the Web as a globally interlinked repository of HTML-formatted documents, we can also see the Web as a globally interlinked repository of data assets that rely on other kinds of markup language. This provides us with an alternative vision of the Web, one which has come to be known as the ‘Linked Data Web’ or the ‘Web of Data’ (see Heath and Bizer 2011). Other kinds of ‘Web’ are also beginning to emerge based on the kinds of resources that are accessed using HTTP mechanisms and URI naming schemes. In addition to the Web of Data, for example, recent work in Web and Internet Science (WAIS) has focused on the Semantic Web (see Shadbolt et al. 2006) and the Web of Things (WoT) (see Guinard et al. 2011). Given the diversity of resources in play here, it should be clear that what makes something a part of the Web has nothing to do with its actual nature (e.g. its material composition). Rather, what is important is the way in which a particular resource is identified and accessed within the larger technological infrastructure of the Internet.

Now that we have a better understanding of what the Web is, we are in a position to specify what is meant by the notion of a ‘Web-based system’. Following on from the aforementioned definition of the World Wide Web, we can define a Web-based system as follows:

(**Web-Based System**) A Web-based system is any system in which at least some of the constituent elements are Web resources (i.e. resources that form part of the Web).

A Web-based system is thus a system that consists, at least in part, of one or more Web-based resources, i.e. resources that are identified using URIs and accessed using HTTP. This definition enables us to treat many kinds of systemic organization as Web-based systems. In some cases, we can view Web-based systems as purely technological systems, consisting of (e.g.) physical devices, software components and digital encodings. At other times, we can view Web-based systems as materially heterogeneous ensembles involving one or more human agents. It is in virtue of this sort of conceptualization that we can now begin to creep up on the notion of a Web-extended mind. For a Web-based system that includes one or more biological entities is already a form of bio-technological ensemble that participates in complex forms of (physically distributed) information processing. All that needs to happen in order for us to recognize such systemic organizations as Web-extended cognitive systems (or Web-extended minds) is for the biological and technological elements of the relevant system to participate in processes that we recognize as, in some sense, cognitive in nature. Of course, the issue of what it is that makes a process cognitive (as opposed to non-cognitive) is something that remains a point of contention in the philosophical community (e.g. Rowlands 2009;Adams and Aizawa 2010). In spite of this, I suggest that for the purposes of getting a firmer grip on the notion of a Web-extended mind, we should set such issues to one side and accept that our pre-theoretical intuitions can serve as a reliable guide as to what it is that makes something a cognitive process.^3^

The result of all this is that a Web-extended mind can, for present purposes, be defined as follows:

(**Web-Extended Mind**) A Web-extended mind is an extended cognitive system whose processes supervene on a set of constituent material elements that includes one or more Web resources.

**Fig. 1:**
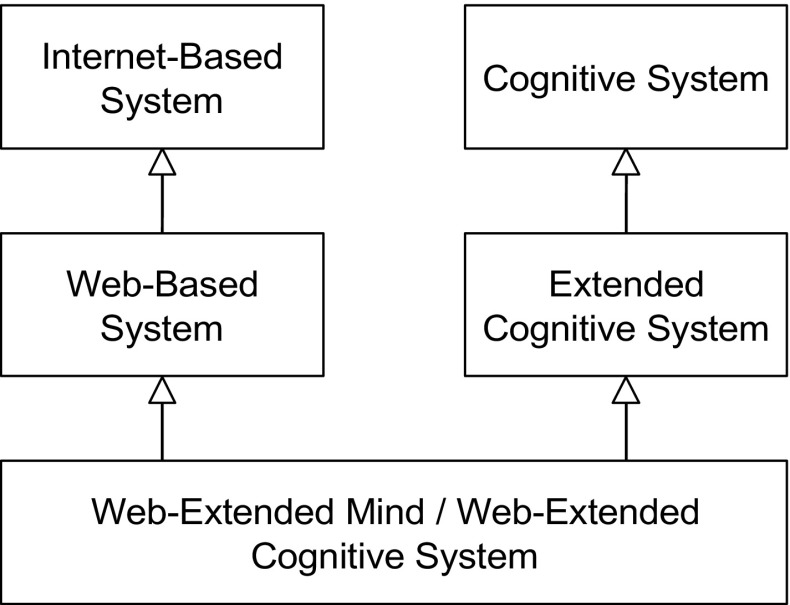
The notion of a Web-extended mind (or Web-extended cognitive system) is defined as a sub-type of both an extended cognitive system and a Web-based system. A Web-based system is, in turn, defined as a sub-type of an Internet-based system, while an extended cognitive system is defined as a sub-type of a cognitive system. Note that the arrows in this diagram symbolize sub-type relationships. Given the nature of the taxonomic relationships in play here it should be clear that a Web-extended cognitive system is also a type of Internet-extended cognitive system

In other words, a Web-extended mind is a systemic organization that exists as both a Web-based system and an extended cognitive system (see Fig. 1).

### Availability

With a clearer sense as to what is meant by the notion of a Web-extended mind, we can now turn to the issue of whether the current (or near-future) Web is able to yield a plentiful supply of extended cognitive systems. As was mentioned above, one way of making progress on this issue is to refer to the criteria proposed by Clark and Chalmers (1998) (i.e. the criteria of availability, trust and accessibility). The first of these criteria concerns the availability of the resource that serves as a source of information. According to this criterion, one of the things that makes a bio-external resource apt for cognitive incorporation is the fact that it is ‘reliably available’ and ‘typically invoked’. This seems to suggest that issues of portability and mobility are relevant to cognitive extension: the more portable something is, the better its candidacy for cognitive incorporation. In this respect, the general thrust of technology development would seem to speak in favor of Web-based forms of cognitive extension (see Smart in press). This is because when we look at recent developments in mobile computing, it seems that a broad array of highly portable devices (e.g. smartphones) are able to provide ever more convenient ways of accessing the online environment. Although some have expressed reservations about the power dependencies of these devices (see Heersmink 2012), it seems as though the current portability of a typical smartphone—the apotheosis of mobile device design—is probably not all that dissimilar to the notebook resource that features in the classic (Otto) thought experiment discussed by Clark and Chalmers (1998). For this reason, we might be inclined to see the smartphone as a device that is suitably poised, under the availability criterion, to participate in extended nexuses of cognitively relevant information processing.

There is, however, an important issue that comes to light when we focus our attention on portable, Internet-enabled computing devices. This concerns the way in which a mobile device is used to gain access to some body of *online* information. Here, we encounter an important difference with the Otto notebook case. In the case of Otto’s notebook, it clearly makes sense to talk of the notebook as a container for belief-relevant information—the encodings that Otto relies on are, we can assume, neatly stored on the individual pages of his notebook. The mobile device, however, does not submit to this sort of characterization. It does not, therefore, seem appropriate to say that in carrying a mobile device around with us we are also transporting the physical vehicles that realize our body of bio-external beliefs. This becomes all too apparent in cases where wireless access to the Internet is denied. In such disconnected scenarios, the device is no longer able to guide our thoughts and actions in a way that is sufficient to legitimate claims regarding states of extended (dispositional) belief.

This seemingly trivial observation has a number of important implications for extended mind theorizing. Firstly, note that in the mobile device case it is no longer appropriate to talk of information being *contained in* a resource. This, recall, was how the third of the trust and glue criteria—the one relating to accessibility—was presented (see above). Secondly, it should be clear that what counts in the mobile device case is not so much the availability of the device that is used to access a body of online information; instead, what matters is the availability of the online information itself! A disconnected smartphone might thus be next to useless in terms of its ability to function as an extended mind resource, especially one that helps to bring Web-based forms of cognitive processing into existence.

When it comes to issues of availability, therefore, what seems to be of primary importance is not the availability of the resource that is used to mediate access to information (the notebook or smartphone); rather, what matters is the availability of the information itself. In the Otto case, this issue does not arise because the notebook and its informational encodings are co-located. In the Web-extended mind case, however, the availability of the information *does* matter because the mere availability of the device is not (by itself) sufficient to secure access to a body of belief-relevant information.^4^

This highlights a potential problem with the availability criterion in its current form. In particular, the availability criterion emphasizes the availability of the physical resource (e.g. the technological device) that is used to deliver information. This seems perfectly appropriate in the classic Otto case, since the distinction between resource availability and information availability is of nugatory significance: the relevant body of information is always stored in Otto’s notebook and thus wherever the notebook goes the information is sure to follow. This is not the case with an Internet-enabled device. In this case, the technological device could be readily available (and thus meet the availability criterion); however, it could still fail to provide access to the relevant body of online information, perhaps because its networking capabilities have been temporarily disrupted.

The upshot of all this is that the availability criterion looks to be in need of revision. In particular, we should perhaps insist that in addition to a resource being reliably available, it should also be suitably poised to provide access to some relevant body of bio-external information. But now notice something important: once we accept the dissociability of an extended mind resource and its informational content, the need for a separate availability criterion is itself called into question. This is because claims about the availability of information content do not appear to buy us anything over and above what is already established by the accessibility criterion. The accessibility criterion, recall, already assumes that some body of information will be available to an agent, and thus an appeal to the accessibility criterion seems able to do much of the work that is deemed to be within the remit of the availability criterion.

A further reason to doubt the need for a separate availability criterion is revealed once we direct our attention to issues of device independence. There may thus be a variety of ways in which we are able to access online information via contemporary digital devices. We can obviously access a particular body of online information via a conventional Web browser while seated at a desktop computer. However, we may also access *exactly the same body of information* via a data-driven smartphone app, a wearable device or an object that exists as part of the Internet of Things (IoT) (Miller 2015). Given the multiplicity of ways in which our access to online content can be mediated, we can (and should) ask whether it still makes sense to talk about a *particular* resource being ‘reliably available’, or even the need for it to be ‘typically invoked’. Such ‘availability-oriented’ intuitions perhaps arise as a result of the current philosophical preoccupation with the Otto notebook case. Inasmuch as this is true, it seems as though philosophical progress in this area may have been somewhat stymied by the understandable enthusiasm and interest that has been generated by the Otto thought experiment. This, I suggest, is one reason why it helps to direct our attention to the possibility of Internet-extended cognition. By considering cases of Internet-extended cognition, we are able to reveal a range of issues that would otherwise be hard to discern and thus likely to escape philosophical scrutiny.

### Trust

The trust criterion has turned out to be one of the more problematic criteria confronting the Web-extended mind hypothesis (Smart in press; 2012). Clark, for example, suggests that the Internet is an unlikely candidate for cognitive incorporation on the grounds that it falls foul of the trust criterion. In particular, Clark suggests that access to Google (even a form of mobile access) is not sufficient for cognitive extension on the grounds that Google-derived information is subject to a form of critical evaluation (see Clark 2010b, p. 46). Clark is, of course, talking about a particular kind of Internet access here. It is highly unlikely, I suspect, that he would seek to champion the view that absolutely *no* form of contact with the Internet could ever (at any time) count as a form of extended cognizing. In fact, what seems to be fueling Clark’s unease with the Internet is tied to the fact that we often have very little control over what appears online. Given that much of the information we encounter on the Web is generated by other agencies, it seems reasonable to assume that such information is treated in a manner that is profoundly different to that encountered in the case of internally situated (i.e. brain-based) information flows. Based on this sort of disparity, it is perhaps hard to see external, Internet-involving informational circuits as functioning in roughly the same sort of way as those that exist solely in the intra-cranial domain.

In evaluating the extent to which the trust criterion poses a legitimate threat to the possibility of Web-based forms of cognitive extension, there are, I suggest, a couple of issues that should be the focus of future scientific and philosophical attention. Firstly, we should ask to what extent contemporary Web users really do approach online information in the sort of way that is deemed inimical to extended mind accounts. Do Web users really subject online information to the sort of critical evaluation that would undermine their status as Web-extended cognizers? Although it might seem appropriate, on epistemic grounds, to be somewhat circumspect about what appears on the Web, why should we assume, in the absence of empirical confirmation, that Web users really do behave in this sort of epistemically optimal manner?

Secondly, we should ask what is really implied by the trust criterion. This is important, because a casual reading of the trust criterion might lead one to the conclusion that *any* form of evaluation and endorsement is off limits. In fact, in his analysis of the extended mind thesis, Michaelian (2012a) questions the extent to which we should see bio-external forms of memory as a genuine form of memory given a particular reading of the trust criterion. One reason for scepticism about extended memory, Michaelian argues, is based on the idea that no form of endorsement of bio-external information is permitted in the case of extended minds. This, Michaelian suggests, undermines the very notion of extended/external memory, since biologically based forms of mnemonic retrieval are, in fact, subject to evaluative assessment:

...due to the constructive character of encoding, consolidation, and retrieval, [mnemonic] records are not endorsed automatically upon retrieval–metamemory processes rather intervene to determine [the] endorsement/rejection of retrieved records. (Michaelian 2012a, p. 1156)

This is where it pays to subject the trust criterion itself to critical evaluation and careful scrutiny. For the idea that *no* form of critical assessment is permitted in the case of extended cognition is not, I think, what Clark is attempting to guard against. This becomes clear when we consider what Clark has to say in respect of the epistemic evaluation of notebook information in the Otto case:

But the information in the notebook, when the notebook is invoked in a bout of active problem-solving, is subject to all the *automatic sub-personal checks and balances that apply to information retrieved from bio-memory*. These checks and balances...in no way require that the notebook be encountered, or even be poised to be encountered, by the agent as an object of active epistemic scrutiny. (Clark 2015, p. 3770) [emphasis added]

Here, we can see that Clark accepts the possibility that *some* form of evaluative assessment is entirely permissible in the case of extended cognitive systems. What he is trying to guard against is the idea that these forms of evaluative assessment are radically different to those that operate in the case of brain-based information flows.

When it comes to issues of trust, therefore, it is important to determine whether (and to what extent) individuals subject online information to forms of vetting and validation that are distinct from those that operate in the internal, neural realm. This, it seems to me, is as an issue that requires as much empirical attention as it does philosophical analysis. Indeed, I suspect that the issue is not one that can resolved solely from the comfort of the philosophical armchair.

### Accessibility

Finally, how does the Web fare when it comes to issues of accessibility—the third of Clark’s (2010b) trust and glue criteria? There are, it seems, conflicting views here. A somewhat negative view is expressed by Smart (2012). He claims that the nature of the informational contact we have with conventional HTML Web pages is (for the most part) inadequate with respect to issues of accessibility. One reason for this, he claims, is that relevant information is often embedded in much larger bodies of online content. The result is that we often have to invest considerable time and effort retrieving relevant information.

**Fig. 2:**
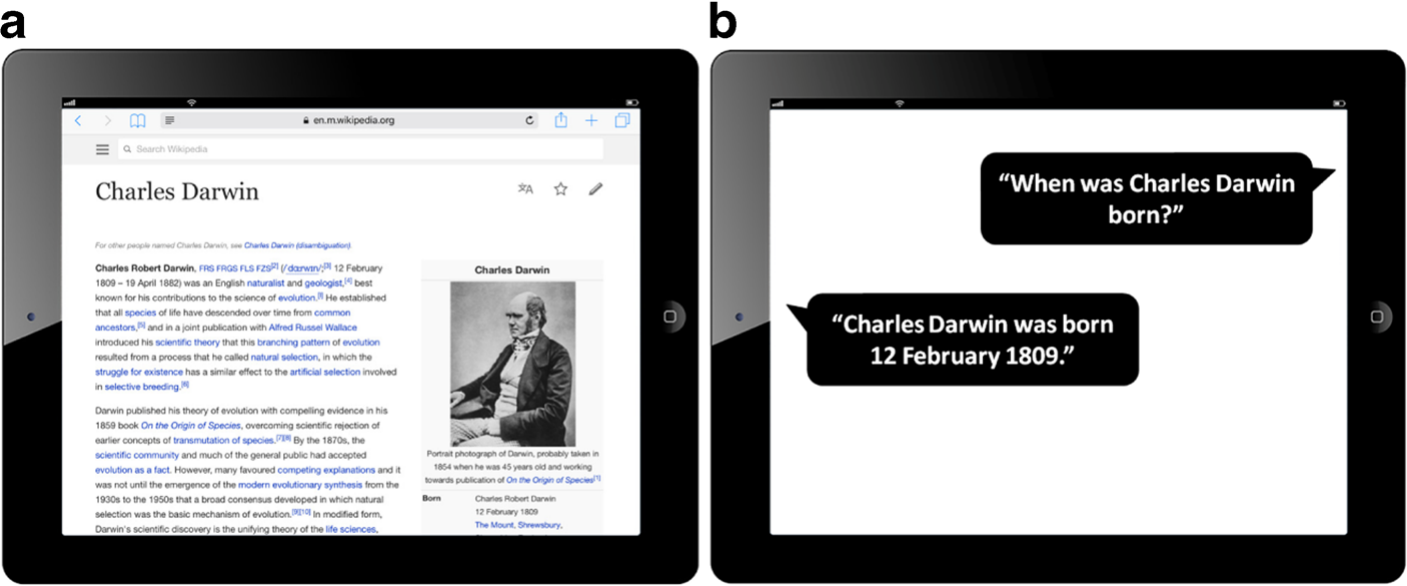
Two screenshots showing information relating to Charles Darwin’s date of birth. **a** The Wikipedia entry for Charles Darwin, as viewed on an Apple iPad device. **b** The use of Apple’s Siri agent to access the relevant item of information

A more upbeat assessment of informational accessibility on the Web is provided by Ludwig (2015). He suggests that even in the case of conventional Web pages—the sort of resources targeted by Smart (2012)—we are still able to enjoy the sort of access that motivates claims regarding extended realization bases for states of (e.g.) dispositional belief. Ludwig, in fact, relies on this to substantiate claims regarding the potential explosion of beliefs and knowledge that occur as a result of our typical (browser-based) forms of contact with the online realm (see Section 5). Ludwig (2015) thus suggests that if we want to ‘know’ the birth date of Charles Darwin, we can simply access the relevant Wikipedia article and find the required piece of information conveniently located at the top of the page. It is thus the location of the relevant information item—the fact that the birth date appears at the top of the page, as opposed to being deeply embedded within the target article—that determines whether it is a candidate for cognitive incorporation (see Fig. 2a).

Unfortunately, I do not think that Ludwig’s claims about accessibility can be made to work. It seems to me that the ‘location’ of an item of information within a particular resource is liable to change as the focal resource is subject to communal edits, or as the resource is viewed on different devices.^5^ It is this locational lability, I suggest, that undermines Ludwig’s attempt to defuse claims regarding the (in)accessibility of online information.

All, however, is not lost; for a recent focus of research attention in the WAIS community concerns the development of new representational schemes for the Web. There has thus been a recent attempt to move towards a more data-centric vision of the Web, one which emphasizes the role of the Web in providing access to globally distributed bodies of machine-readable information (Wood et al. 2014; Bizer 2009; Heath and Bizer 2011). Crucially, the kinds of representational formats that feature as part of this ongoing research and development effort are ones that promise to transform the nature of our informational (and perhaps cognitive) contact with the Web. Linked data resources are thus able to serve as a back-end repository for all-manner of data-driven apps and services, many of which can be tailored to provide easy access to specific bodies of information content. The use of online data assets also helps to free us of the otherwise restrictive notion that all cognitively relevant information in the online realm needs to appear in textual form. Data-oriented representations thus enable information (sometimes the same information) to be presented in a variety of different ways, for example, as verbal prompts, graphical cues, and augmented reality overlays (see Smart 2014).

The Web of Data not only promises to reshape our traditional approach to accessing online information, it also opens up a wealth of opportunities to develop various forms of ‘assistive intelligence’. In order to help us see this, think about the way in which a body of semantically enriched online data (see Shadbolt et al. 2006) might be exploited by a speech-enabled intelligent assistant, such as Apple’s Siri or Microsoft’s Cortana. Suppose that the body of data being exploited relates to the birth dates of significant historical figures. Given that the data is both machine-readable and annotated in ways that supports various forms of (semantically enabled) search and reasoning, it now becomes possible to initiate new forms of epistemically potent interaction with a conversationally enabled digital companion. You can thus ask Siri questions, such as ‘When was Charles Darwin born?’ Almost immediately, you will hear the response: ‘Charles Darwin was born the 12th February 1809’ (see Fig. 2b). This, at least, is the response that was provided by my own Siri-enabled device at the time of writing.

As a further example of this sort of assistive intelligence, imagine that you are walking in the forest with a friend. As usual, you are equipped with a portable smartphone device. In addition, you are wearing a head-mounted augmented reality display device (a future version of Google Glass perhaps) that is able to interact wirelessly with your smartphone and present Internet-accessible information directly within your field of view. Suddenly, you and your friend come across an odd looking tree, one that appears different from all the rest. ‘Do you know what kind of tree that is?’, asks your friend. ‘Sure’, you say. You hold up one of the leaves from the tree in front of the camera embedded in your headset. The headset transfers the image to your smartphone, which performs some preliminary image analysis and identifies the object as a leaf. The phone then invokes the services of a tree classification app,^6^ which analyzes the image, extracts some relevant features and acquires contextual information relating to (e.g.) your geographic location. The app then proceeds to query an online linked data repository containing information about the relationship between tree species and their associated characteristics. The name of the tree species is subsequently returned to the app and ultimately transferred to your headset. The headset, in turn, displays the (correct) name of the tree species directly within your field of view.

An important question to ask at this point is whether it is appropriate to conclude that you really do ‘know’ the relevant tree species, perhaps even before you recruited the services of your arsenal of ambient technological aids. Do you qualify as an arboreal classification expert, despite the fact that quite a lot of the relevant processing seems to be taking place beyond the borders of the biological domain? Perhaps, as is often suggested in cases of extended cognition, the processing loop that travels through your smartphone and into the online environment should be considered as functionally equivalent to the brain-based neural circuits that would otherwise enable you to retrieve information from bio-memory. Inasmuch as this is indeed the case, why should we seek to cast aspersions on your arboreal expertise? If online information is poised to influence your thoughts and actions in roughly the same sort of way as information retrieved from bio-memory, then what is the basis for claiming that cases of brain-based information retrieval are indicative of a particular kind of *cognitive* competence, whereas the retrieval capabilities of the headset-phone-app-Internet ensemble fail to hit the (cognitive) mark?

We can now see, in the wake of these two examples, how the transition from a document-centred Web (populated by conventional Web pages) to a data-centred Web (populated by a rich and interconnected array of linked data assets) could transform the kinds of cognitive contact that we are able to establish with the online environment. The general moral here is that we should not confuse the shortcomings of one particular form of Web access with the properties of *all* forms of Web access. Given the popularity and ubiquity of conventional (HTML) Web pages, it is easy for philosophers to be misled into thinking that such resources are the only kinds of resources that are relevant to issues of cognitive incorporation. The technological remit of the Web, however, is much broader than conventional Web pages, and such resources certainly do not exhaust the range of interactive opportunities that are likely to be available to the human user community (both now and in the future). When it comes to the accessibility of online information, then, there is a pressing need for philosophers to broaden the scope of their current analytic efforts.

## Over-Extended Minds?

As we saw in the previous section, the nature of our relationship with the contemporary Internet seems to allow for the possibility of various forms of Internet-extended cognition. This positive assessment is further reinforced, some have claimed, once we consider the general trajectory of technology development (see Smart in press). In fact, the nature of emerging digital technologies may mean that the Internet-extended mind is able to serve as a better example of cognitive extension than the original Otto case. Smart (in press), for instance, suggests that an online economy of machine reasoners and semantically enriched content is able to support the kind of informational updating and inferential coherence that is sometimes deemed to be of crucial relevance to the doxastic status of extended cognizers (Weiskopf 2008; Wikforss 2014). This is not to say, however, that Internet-based forms of cognitive extension are entirely unproblematic, or even that they do not give rise to issues that might be seen as inimical to the very idea of extended cognition. In what follows, I attempt to identify a number of areas where a consideration of Internet-based forms of cognitive extension might serve as a source of grist for the sceptic’s mill.

### Network-Extended Cognitive Bloat

One of the problems afflicting extended mind accounts is the notorious problem of ‘cognitive bloat’ (Allen-Hermanson 2013). Cognitive bloat refers to an unwelcome expansion in the sorts of things that count as part of the machinery of the mind. As is noted by Allen-Hermanson (2013):

While EC’s [extended cognition’s] advocates wish to ‘supersize’ the mind, they have no desire to overextend the boundaries. All sorts of things that are not part of one’s mental vehicle play a causal-explanatory role when it comes to supplying information. If a notebook counts as part of one’s mind, then why not the yellow pages, the internet, or even parts of the natural world that supply information and support cognition? (Allen-Hermanson 2013, p. 792)

Issues of cognitive bloat typically arise in respect of particular kinds of resources, such as the much maligned *Encyclopaedia Britannica*. The nature of these resources is deemed to be so at odds with our intuitions as to what can serve as a genuine realizer of cognitive states and processes that they are seen to lead to a very *reductio* of the idea that the mind extends beyond the biological borders of the individual.

In the case of Internet-based forms of cognitive extension, it is possible to identify a somewhat different kind of cognitive bloat. This kind of bloat stems from the fact that the Internet is a global information and communication network that supports highly distributed forms of information access, data representation and service invocation. In applying notions of cognitive extension to the Internet, we thus come face-to-face with the idea that the vehicles of cognition might be situated some physical distance from the individual whose cognitive apparatus is supposedly extended. In the conventional case of Otto and his notebook, this sort of distance-related issue does not arise. This is because Otto’s notebook and all its doxastically relevant encodings are located within the immediate vicinity of Otto. But now imagine a technologically upgraded version of the Otto case, where the notebook is replaced with a smartphone and the contents of Otto’s notebook are stored in the cloud, perhaps in an Internet-accessible database that is located on the other side of the planet. In this case, our active externalist intuitions are arguably irritated by issues of physical distance and distribution. Should we really accept the claim that a resource located on the other side of the planet is able to function as part of an extended cognitive circuit? When we think about extended cognitive systems, it is natural to think of a nexus of materially heterogeneous resources that are linked together by cognitively relevant forms of causal commerce (see Smart et al. 2010). But just how far should we allow that network to extend beyond the biological borders of the individual? Providing one accepts that Otto and his notebook do indeed count as a *bona fide*case of cognitive extension, then the appeal to more technologically sophisticated scenarios (e.g. those involving a neurologically normal individual equipped with a smartphone) may look to be largely innocuous. However, the case of Internet-extended cognition gives rise to issues that were not apparent in the original Otto case, and some of these issues may work to offend the intuitions of even the most ardent advocate of active externalism. In the current case, we have to ask ourselves at what point (if any) should we seek to draw a line regarding the spatial extent of the network that realizes a putative case of extended cognizing. The local interactions between an individual and their smartphone do not seem to pose much of a problem here. But what about the larger representational and computational nexus that enables and supports that local interaction? Should a set of remotely located servers, circuit boards and databases *also* count as candidates for cognitive incorporation, in addition to the more proximally situated smartphone device?

The main point, then, is that a consideration of Internet-extended cognition seems to lead to a concern about the physical (spatial) distribution of the bio-external resources that feature as part of an extended cognitive system. This issue is not so readily apparent in the more conventional cases of cognitive extension (e.g. the Otto notebook case), because many of those cases do not feature the involvement of resources that are physically remote. When we focus on a paper-based notebook as an extended mind resource, for example, issues of physical (spatial) distribution do not arise. This is because when the notebook is physically remote, it is no longer a candidate for cognitive incorporation on account of the fact that it is no longer accessible to the relevant individual. It is, in fact, only when the notebook is within ‘touching distance’ of the individual that we deem the notebook to be of (potential) constitutive relevance to the individual’s cognitive economy.

Perhaps one way of making progress on this issue is to revisit a thought experiment that was originally proposed by Daniel Dennett (1981) in a paper titled ‘Where am I?’. Let us therefore imagine a situation in which some part of an individual’s brain—their hippocampus, let’s say—was anatomically separated from the rest of their brain and transported to the other side of the planet. Now let us assume that the isolated hippocampus was suspended in a nutritive jar of fluids in order to keep it ‘alive’, and that it was re-connected to the rest of the individual’s brain using a combination of neural interfacing techniques and Internet-mediated forms of information transfer. In this case, the Internet serves as part of the physical network that allows for the bidirectional flow of signals between the physically remote hippocampus and the rest of the individual’s biological brain. Although this case is entirely fictional, we can use it to pump our intuitions concerning the role of physical distance in undermining claims for cognitive extension. We can thus ask whether we would be content to view the remotely located hippocampus as part of the physical machinery of the mind if it was seen to support the expression of intelligent behavior in more-or-less the same sort of way as it did when it was located inside the skull. If we are able to answer in the affirmative here, it is unclear why issues of physical distance, by themselves at least, should count against the possibility of Internet-based forms of cognitive extension.

### Local vs. Remote Resources

One way of responding to worries about network-extended cognitive bloat is to limit the set of non-standard physical realizers to those that are located in the immediate vicinity of the biological individual. Accordingly, we might insist that in order for something to count as a candidate for cognitive incorporation it needs to be in direct contact with the sensorimotor surfaces of the human individual. Anything that fails to meet this criterion, we might insist, should not count as part of the realization base for an individual’s cognitive states and processes. In this case, an Internet-enabled smartphone would still count as a candidate object for cognitive extension, as would the information that is provided by the smartphone (following its retrieval from some remotely located database or website). What would not count as a candidate for cognitive incorporation, however, are those elements that are distal to the sensorimotor surfaces of the biological agent, namely, the remotely located database and its associated informational content.

As a means of tackling issues of network-extended cognitive bloat, this sort of ‘spatial proximity criterion’ looks to be highly effective. In addition, the criterion looks to be rather innocent from an extended mind perspective. In particular, by embracing the criterion, we are not ruling out the possibility of cognitive extension; we are merely stating that there should be some form of direct contact between the biological individual and the set of resources that function as the constitutive elements of an extended cognitive circuit.

**Fig. 3:**
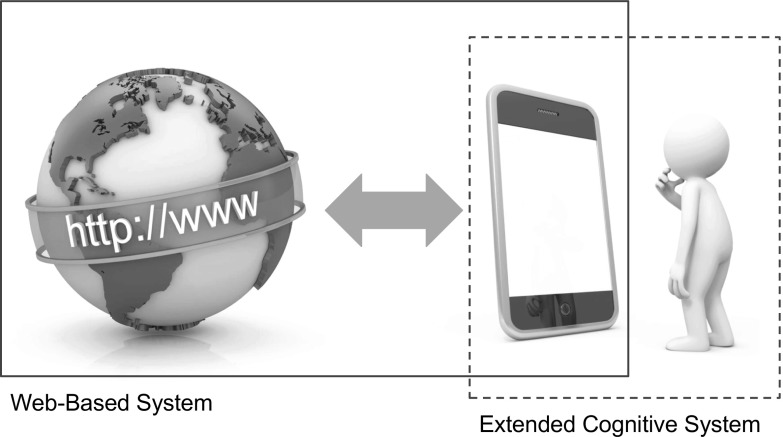
The imposition of the spatial proximity criterion means that the boundaries of an extended cognitive system no longer reach out to include a set of Web-based resources. The result is that we have two systems—a Web-based system and an extended cognitive system—however, the extended cognitive system no longer counts as a Web-based system. This is sufficient, it seems, to rule out claims of Web-extended cognition under the terms of the definition proposed in Section 2.1

Unfortunately, from the perspective of Internet-based forms of cognitive extension, the spatial proximity criterion is far from innocent. In fact, inasmuch as we accept the criterion, then the prospects for perhaps *any* form of Internet-based cognitive extension starts to look a little bleak. This is because by limiting the reach of the extended mind to those regions of the physical environment that are in more-or-less direct contact with the sensorimotor surfaces of the biological individual, we rule out the possibility of including online, Internet-accessible resources within an extended cognitive organization. In order to help us see this, recall the earlier discussion regarding Web-extended minds and Web-based systems (see Section 2.1). Recall that as part of that discussion we encountered the claim that what makes something a Web-extended mind is the fact that it can be regarded as a specific form of Web-based (cognitive) system; i.e. a system that includes one or more Web-accessible resources (where the notion of a Web-accessible resource is bound up with the use of Internet application protocols and resource identification schemes). This definition provides, I suggest, a reasonable guide as to what counts as a Web-extended mind. But note that once we limit our attention to those resources that are located in the immediate vicinity of the biological body, it is by no means clear that it still makes sense to talk of extended cognitive circuits as ones that include Web-based resources. Crucially, the nature of an individual’s informational contact with an Internet-enabled device (a smartphone, let us say) is mediated primarily by their tactile, visual, auditory and motor output systems. But in establishing contact with the smartphone, these biologically based sensor and effector systems do not establish any form of *direct* contact with a Web resource (i.e. they do not feature the use of HTTP mechanisms and URI naming schemes). An individual’s immediate (sensorimotor) contact with the smartphone is, as a result, not one that can be glossed in Web-related terms—the resources in question are not *sensu stricto* Web resources. Clearly, in the process of retrieving information from the Web and presenting it to an agent via a display screen, the smartphone itself is required to engage in a form of interactive engagement with the Web, and this may very well warrant talk of the smartphone as (at least temporarily) existing as a constituent element of a Web-based system. However, it is by no means clear that it still makes sense to see the biological individual as a component of that system. Instead, what we seem to have here are two systems that intersect in the region of an Internet-enabled device (see Fig. 3). One of those systems is (on occasion) an extended cognitive system; the other system is (on occasion) a Web-based system. But it does not seem appropriate, under the spatial proximity criterion, to see the extended cognitive system as itself a form of Web-based system. There is, in other words, no reason to see Web resources as an intrinsic part of the causally active physical fabric that realizes an individual’s cognitive states and processes. It is in this sense that the spatial proximity criterion is, I suggest, inimical to the idea of Web-extended (or, more generally, Internet-extended) cognition.

In opposition to this rather dismal conclusion, proponents of the extended mind might want to point out that our sense as to what should count as the sensorimotor surface of a cognitive agent is itself something that is challenged by the notion of cognitive extension. If we are indeed entities that are subject to various forms of cognitive and bodily extension (see Clark 2007), why should we continue to privilege the biological borders of the individual in staking out what counts as a constituent element of a cognitive system: if my body and mind are extended by the addition of some kind of technological prosthesis, such as an iPhone, why should my sensorimotor surfaces not extend to encompass the capabilities of the technological prosthesis itself? Why, in other words, should the interactive capabilities of the (cognitively integrated) iPhone not be counted as part of the sensorimotor paraphernalia that I, as a cognitive agent, rely on to help me navigate the cognitive and epistemic terrain of the online, digital world? As a means of further pressing this case, consider again the hippocampus-in-a-vat scenario (see Section 3.1). There is clearly a sense in which the remotely situated hippocampus counts as an Internet-accessible resource. It is thus a resource that engages in rich bidirectional forms of Internet-mediated communication with the rest of the biological brain, and such bidirectional communication occurs as a result of (let us suppose) the use of a specific (perhaps bespoke) Internet application protocol. There is also a sense in which the nature of an agent’s contact with the remote hippocampus is at least somewhat indirect. There are, we may assume, multiple kinds of switching, gating, routing, signalling and intermediate computational processing that occur every time the hippocampus is called on to service the representational and computational needs of the rest of the (brain-based) cognitive economy. The critical question that gets raised by all this is the following: why, if we are happy to accept that the hippocampus-in-a-vat counts as part of the machinery of the mind, should we discount Internet-accessible resources as candidates for cognitive incorporation solely on the basis that they are not within ‘direct’ reach of our biologically based sensorimotor systems?

### Cognitive Cabling

Another line of attack for those who are sceptical of claims regarding Internet-extended cognition concerns the role played by physical cabling in supporting the flow of information across the Internet. Surely, a sceptic might insist, we do not want to allow that a lengthy stretch of cable should count as part of the machinery of the mind simply because we happen to rely on the information contained in a physically remote Web resource. If we accept the idea that cabling should count as part of the physical machinery of the mind, won’t we risk committing ourselves to the seemingly ludicrous idea that a trans-oceanic telecommunications cable is, at some time or another, a constituent element of a vastly (over-)extended cognitive system?

Echoes of this sort of worry can be found in the mainstream extended mind literature. In their critique of the notion of extended cognition, for instance, Adams and Aizawa (2008) suggest that the mere transfer of information (from one location to another) is not sufficient to warrant claims regarding cognitive incorporation. This, they suggest, applies as much to the inner, neural realm as it does to the world beyond the organismic boundary. On their view, the corpus callosum should be regarded as a passive carrier of information—a ‘mere bus’— rather than as some active element of the neural information processing economy (see Adams and Aizawa 2008, p. 17). This kind of information-transfer-related worry is, it should be clear, simply the neurological counterpart to the worry about Internet cabling. Such cabling, it might be said, undoubtedly plays an important role in supporting the run-time realization of cognitively relevant information processing routines, but it does not—at least to any significant extent—participate in the active manipulation and transformation (i.e. the processing) of such information. As a result, Internet cabling should not count as part of the realization base for human mental states and processes.^7^

By way of responding to this worry, we might want to question the extent to which we can actually disentangle issues of ‘mere information transfer’ from issues concerning the mechanistic realization of cognitive processes. In fact, as is noted by Clark (2010c), it is far from clear that we can perform the required separation; neither is it clear that we even make sense of the idea that the pattern of information flow, as enabled by some set of connective elements (e.g. cabling), is utterly irrelevant to the project of understanding the operation of an information processing system. In the case of the biological brain, for example, the nodal elements (i.e. neurons) are elements that participate in both the processing of information as well as the transfer of information to remote anatomical regions via their axonal projections. Should we thereby insist that *only* the cell body of the neuron should count as cognitively relevant, while all the axonal elements are to be excluded? This, to my mind, is nonsensical. When it comes to the biological brain, as well as an array of other networked systems, what really matters to information processing is the pattern of information flow and influence that is enabled by the structural organization of the system. In other words, it is the time-variant topological structure of the information processing network that plays a crucial role in enabling us to understand the properties (cognitive or otherwise) of the larger systemic organization.^8^ Given that this structure is determined by the set of physical linkages that connect one element to another, why should we fail to appreciate the *cognitive* significance of the connective elements when the focal system is recognized as a *bona fide* cognitive system. To do so is surely to overlook an important body of scientific work that emphasizes the role of network structure in enabling us to attain an explanatorily potent grip on the behavior of a broad array of materially diverse complex systems (Buchanan 2002; Barabasi 2002; Baronchelli et al. 2013).

## Intimate Connections

**Fig. 4:**
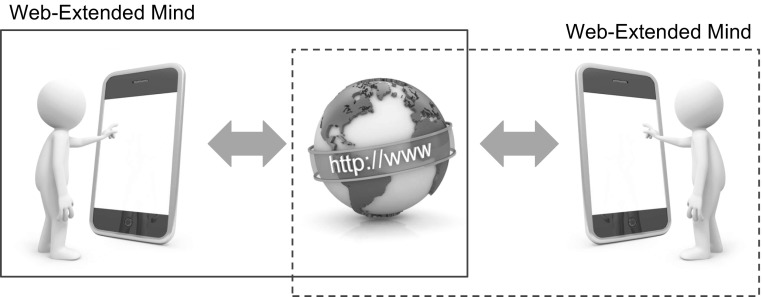
The notion of conjoined minds refers to a situation in which some online, Internet-accessible resource serves as a common element of two (or more) Internet-extended cognitive systems (in this case, Web-extended minds)

Perhaps one of most distinctive features of the Internet is its connectivity—the fact that it permits almost anyone to share and access information, pretty much from anywhere in the world. This feature marks the Internet out for special consideration when it comes to issues of extended cognition. This is because the Internet forces us to consider cases in which the supervenience base for one individual’s mental states and processes could be seen to overlap with that of another individual. To help us gain a better understanding of this idea, consider a situation in which two or more individuals access the same piece of online information as part of some episode of extended cognitive processing. Imagine, for the sake argument, that two individuals use their smartphones to retrieve information about the location of the Museum of Modern Art. If we restrict our attention to just one of these individuals, the situation appears to resemble the classic case of Otto and his notebook. Indeed, inasmuch as we accept the basic idea of Web-based forms of cognitive extension (see Section 2), then it seems appropriate to see the online resource as part of the mechanistic supervenience base for the focal individual’s dispositional beliefs about the museum’s location. But now notice what happens when we broaden our analytic gaze to include the second individual. In this case, it becomes apparent that the online resource is a common element in an extended material nexus that comprises the realization base for *both* of the individuals’ location-related dispositional beliefs. In other words, in the case of Internet-extended cognition, we encounter a state-of-affairs in which the (extended) mind of one individual could (on occasion) overlap with the (extended) mind of another individual. In grappling with the implications of Internet-extended cognition, therefore, we have to confront the curious case of what might be called ‘conjoined minds’—the idea that a bio-external resource could form part of the cognitively relevant physical machinery that underlies the mental states and processes of multiple human individuals (see Fig. 4).

The topic of conjoined minds has seldom been discussed in the philosophical literature.^9^ One exception is a paper by Halpin et al. (2010). In regard to Web-accessible content, for example, Halpin et al. hint at the possibility of shared supervenience bases for cognitive states and processes:

Perhaps external representations on the Web, when integrated appropriately into the processes that govern an agents [*sic*] behaviour, may count as parts of that agents [*sic*] cognitive architecture. But now assume that multiple individuals are able to access the same external representation...Here, it seems, more than one person may deserve cognitive credit for, and have cognitive ownership of, a representation that augments their own individual intelligence. (Halpin et al. 2010, p. 2)

One reason why the Internet is of particular importance and relevance here relates to the way in which it affords large-scale social access to specific online resources. It is therefore relatively easy to imagine a state-of-affairs in which multiple individuals are accessing the same body of information at the same time, albeit (perhaps) for different cognitive purposes. This does not mean that conjoined minds are only to be found in situations that involve the Internet—it is, I suppose, possible that conjoined minds could emerge in other kinds of socio-technical context. Nevertheless, the ease with which we are able to entertain the possibility of conjoined minds in an Internet context, as well as the potential scale of such minds,^10^ helps to highlight the philosophical importance of the Internet when it comes to issues of cognitive extension.

## Extended Knowledge

The application of extended mind theorizing to the Internet has potentially profound implications for our status as epistemic agents. If we accept, for example, that online information can play the same sort of functional role as that served by the information in Otto’s notebook, then it seems that the Internet-extended cognizer could be subject to a significant expansion in their body of dispositional beliefs. Indeed, assuming that such dispositional beliefs are true, we may even wonder whether this form of doxastic expansion is sufficient to transform the Internet-extended cognizer into something of a ‘super-sized knower’. The possibility of Internet-extended cognition thus appears to open the door to a profound form of epistemic transformation, one in which the limits of what we know are only bounded by what the Internet makes available (see Smart 2012).

This sort of reasoning forms the basis for a number of recent claims regarding the epistemic impact of the Internet (Ludwig 2015; Bjerring and Pedersen 2014). Ludwig (2015), for example, argues that in the wake of Internet-based forms of cognitive extension, we should anticipate a profound transformation of our doxastic potential. In particular, he anticipates ‘an explosion of dispositional beliefs and knowledge that is caused by digital information resources such as Wikipedia or Google’ (p. 355). Similar views are expressed by Bjerring and Pedersen (2014). They argue that the Web enables us to enjoy various forms of ‘restricted epistemic omniscience’, wherein we have more-or-less ‘complete knowledge about a particular, fairly specific subject matter’ (p. 25).

Are such claims to be believed? Do extended cognizers really give rise to extended knowers? And, if so, what impact does this have on the nature of our individual and collective epistemic capabilities?

In answering these questions, it seems appropriate to turn to our attention to the epistemological literature. A popular way of thinking about knowledge within contemporary epistemology is to emphasize the role played by cognitive abilities in determining the truth status of an individual’s beliefs. An agent *S* can thus be said to know that *p* if and only if *S* believes that *p* and the truth of *S’s* belief that *p* is due to the exercise of cognitive abilities that are ascribed to *S*. This sort of epistemological position is known as virtue reliabilism (Greco 2007; 2012), and it forms part of a more general movement in contemporary epistemology that goes under the heading of virtue epistemology (Greco and Turri 2012).

Relative to the virtue theoretic conception of knowledge, there have been a number of attempts to advance our understanding of what has been dubbed ‘extended knowledge’ (Pritchard 2010; Palermos 2014a; Smart in press). This is a form of knowledge that is (in some way) tied to the operation of extended cognitive routines. There are, however, a number of different interpretations as to what is meant by the term ‘extended knowledge’. One seemingly straightforward option here is to see extended knowledge as a specific form of extended belief. In other words, in making sense of the notion of extended knowledge, we can take the classic case of extended supervenience bases for states of dispositional believing (see Clark and Chalmers 1998) and apply this to the case of beliefs that (*qua* virtue epistemological accounts) qualify as knowledge. Extended knowledge, on this view, is a specific form of materially extended dispositional belief (i.e. a mental state whose realization base extends beyond the traditional organismic boundaries of the biological individual). This, I assume, is the sort of view that is countenanced by both Ludwig (2015) and Bjerring and Pedersen (2014) as part of their attempt to make the case for Internet-enabled forms of epistemic expansion.

There is, however, a problem with this particular view of extended knowledge. Recall that from the perspective of virtue reliabilism, a belief should only count as knowledge if the cognitive abilities of the believer (i.e. the agent that holds the belief) are deemed to play an explanatorily significant role in determining the truth status of the belief. Unfortunately, this is a condition that is utterly inappropriate for the purposes of evaluating the epistemic standing of the Internet-extended cognizer. The reason for this is that the agent that is the subject of the extended belief state (i.e. the candidate knower) is unlikely to be the agent whose abilities (cognitive or otherwise) contributed to the formation of the dispositional belief in question. In most of the cases that Ludwig and others refer to (e.g. cases where an agent is interacting with Google or Wikipedia), there seems to be little reason to think that the subject of the extended belief state plays any (significant) role in the creation of the material resources upon which their dispositional beliefs supervene. As such, it is difficult to see how it is that *their* cognitive abilities could have played any role in determining why it is that *their* states of extended dispositional believing are able to track the truth. In other words, it looks highly unlikely that Internet-extended cognizers can be deemed to be extended knowers, at least from the perspective of a virtue-theoretic account of knowledge.

One response to all this is to claim that the focus of the preceding argument is simply misplaced. Rather than focus on the processes that lead to the formation of a dispositional belief, perhaps it is better to shift our attention to the processes that are in evidence when some body of extra-organismic information is actually exploited by an extended cognitive agent. This seems to be a reasonable strategy; since once we switch our attention to the run-time processes of information retrieval—to the point at which an agent accesses some body of online information—things start to look a lot more positive from the perspective of virtue-theoretic epistemic evaluation. At the very least, we do, in this situation, seem to confront a state-of-affairs in which an agent’s cognitive abilities could be said to be relevant to the truth (or falsity) of the beliefs that they are deemed to hold. In particular, it seems reasonable to suppose that the exercise of an agent’s cognitive abilities are relevant to the appropriate selection and endorsement of particular bodies of bio-external information (Michaelian 2012b). In view of this, we can surely accept that the Internet-using agent believes the truth as a result of the exercise of their cognitive abilities. When it comes to the much vaunted explosion of knowledge associated with the Internet-extended cognizer, perhaps we finally have a means of bridging the apparent gap between extended cognition and extended knowledge.

Unfortunately, however, I do not think things are quite so straightforward. The first thing to note is that in switching our attention away from the processes associated with the formation of dispositional beliefs, we have also moved away from the idea of materially heterogeneous supervenience bases for states of extended belief. In fact, when we focus our attention on the point at which online information is accessed by an agent, it seems that we are no longer talking about dispositional beliefs at all. Instead, it seems much more appropriate to see the processes of interest as leading to the formation of non-dispositional (or occurrent) belief states. There is no reason, to my mind, why we should regard such occurrent beliefs as extended. Rather, it seems that the beliefs of interest are ones that are realized by forces and factors that are wholly *internal* to the biological agent. There does not, in other words, seem to be a compelling reason to see the ‘run-time’ retrieval of online information as leading to the formation of extended beliefs (and thus extended knowledge).

All of this serves to draw attention to some of the complexities associated with the notion of extended knowledge. It also serves to highlight some of the problems confronting claims about the epistemological significance of the Internet (Ludwig 2015; Bjerring and Pedersen 2014). Although it is perhaps natural to assume that Internet-extended cognizers will give rise to Internet-extended knowers, things look a lot more complicated when we attempt to situate claims of extended knowledge within the theoretical frameworks of contemporary epistemology. None of this, of course, should detract from the idea that the Internet expands our epistemic power and potential by serving as an extended cognitive resource. As should be clear from the discussion in Section 2, I tend to think that there are indeed situations where the Internet does lead to a significant expansion in our epistemic capabilities—recall, for example, the case of tree species identification in Section 2.4. To my mind, one of the focus areas for future philosophical work in this area is to develop a better understanding of how to integrate active externalism with (e.g.) virtue theoretic conceptions of knowledge. We should then seek to apply this theoretical synthesis to the specific case of Internet-extended cognition.

## Socially Extended Cognition

For the most part, philosophical treatments of extended cognition tend to limit their scope to systems that involve bio-technological forms of bonding. Another form of cognitive extension occurs, however, in relation to the social environment. In this case, the main focus of attention relates to whether the elements of the social environment (e.g. other human agents) are of constitutive relevance to the realization of an individual’s cognitive processing routines.

There is, of course, little doubt that the social environment plays an important role in shaping our cognitive performances. Consider, for example, the way in which our mnemonic capabilities are often altered as a result of our interactions and engagements with other agents. In the effort to recall the details of some past event, for example, we may engage in a process of collaborative recall, whereby recollective success is mediated by an iterative process of cross-cueing (see Sutton et al. 2010). Similarly, when it comes to the mnemonic encoding of information, our conversational exchanges with others may help to support a form of socially mediated elaborative rehearsal concerning ‘significant’ events and experiences (see Smart 2010).^11^

In the context of debates about the extended mind, the role of the social environment in shaping and supporting our cognitive performances helps to bolster claims regarding socially extended cognition (e.g. Tollefsen 2006). This is a form of cognitive extension that is recognized by at least some proponents of active externalism. In their original treatment of the extended mind thesis, for example, Clark and Chalmers (1998) suggest that there is no reason, in principle, why socially extended forms of cognition should not exist:

Could my mental states be partly constituted by the states of other thinkers? We see no reason why not, in principle. In an unusually interdependent couple, it is entirely possible that one partner’s beliefs will play the same sort of role for the other as the notebook plays for Otto. (Clark and Chalmers 1998, p. 17)

In spite of all this, claims about socially extended cognition remain controversial. This is due, at least in part, to worries about the extent to which other agents can meet the sort of criteria required for cognitive extension, for example, the trust and glue criteria proposed by Clark and Chalmers (1998) (see Section 2).

It is here, I suggest, that a focus on the Internet could be of philosophical value. This is because the Internet plays a crucial role in shaping the nature of our social interactions and engagements, transforming the way in which a broad array of social activities are undertaken. As a result of this transformation, it is possible that the Internet helps to surmount (via technological means) some of the barriers that would otherwise prohibit the practical realization of socially extended cognition. We can thus imagine Internet technologies being specifically designed so as to satisfy the conditions that are deemed to be conducive to socially extended cognition. Such conditions may, of course, be impossible to achieve in the case of conventional face-to-face interactions, and it is for this reason that discussions regarding socially extended cognition are perhaps best situated in the context of the Internet (especially the Social Web).^12^

Not only does the Internet change the nature of our social interactions, it also alters the nature of what it means to rely on the social environment for the purposes of cognitive support. In order to help us see this, consider a number of applications that rely on what is called real-time or continuous crowdsourcing (see Lasecki and Bigham 2013). In general, these applications seek to capitalize on the availability of large groups of individuals in order to support the execution of particular tasks. The VizWiz system, for example, is a system that seeks to support blind people in dealing with the challenges of a visual environment (Bigham et al. 2010). VizWiz enables blind individuals to upload images from a smartphone and then receive descriptions of the image from other individuals in what is described as ‘nearly real-time’. Such systems support the idea that the wider social environment can be recruited into a form of cognitively potent information processing, one which then serves to influence the thoughts and actions of a visually impaired individual. There is an obvious parallel here with the case of Otto and his notebook. Despite some clear differences in the nature of the cognitive processing routines that are being performed (e.g. visual processing versus mnemonic recall), as well as differences in the nature of the relevant material realizers (i.e. socio-technical system versus notebook), the two cases are roughly equivalent: in both cases, we have a form of disability that is being addressed by virtue of the kind of engagements that are made with respect to the extra-organismic environment.

In addition to the issues raised by real-time crowdsourcing and the technological transformation of human social interaction, there is, I suggest, a further way in which the Internet is relevant to the topic of socially extended cognition. The main point of interest here concerns the emergence of technological systems that are intended to function as intelligent personal assistants, conversational partners or even artificial social companions (e.g. Wilks 2010). Systems such as Siri and Cortana are emblematic of the interest that major technology vendors have in supporting new forms of engagement and interaction with the technological domain—especially ones that capitalize on our existing suite of communicative abilities. To an ever-greater extent, such systems are able to emulate the features of human-to-human verbal exchanges. This raises an important issue; for inasmuch as these systems start to resemble the nature of our traditional social interactions and engagements, we can ask whether they might lead to a progressive blurring of the erstwhile crisp distinction between socially- and technologically extended forms of cognition. In other words, as technological systems begin to resemble conventional social partners, perhaps the conceptual distinction between socially extended and technologically extended cognition is itself called into question.

This potential blurring of conceptual boundaries is important when it comes to the way in which the distinction between the social and the technological is used in the context of recent philosophical arguments. Consider, for example, the way in which differences between the social and technological realms are used to promote asymmetric treatments regarding the extendedness of belief-forming processes (Goldberg 2010; 2012). Goldberg (2012) thus suggests that other human agents can form part of an extended belief-forming process but non-human agents (e.g. technological devices and instruments) cannot. This distinction is presumably based on the relative differences between human and non-human agents when it comes to their role as information providers. But once we accept that some kinds of technological system can function in the same sort of way as a conventional human partner, the basis for this distinction looks to be, at best, unclear.

To help reinforce this particular point, suppose that you are walking through the woods with your voice-enabled digital companion and you happen to hear the song of an unknown bird species. Curious about the source of the song, you verbally instruct your digital companion to identify the species of bird responsible for the song. Your digital companion then invokes the services of a birdsong classification program^13^ and promptly communicates the correct answer to you in verbal form. In cases such as these, it is unclear why we should regard the contributions of a digital companion as somehow distinct from those of a human companion. If it makes sense to see one’s belief-forming process as materially extended based on the testimony of a human expert (an ornithologist, let us say), why not see the testimony of a more-or-less constant, trusted and reliable digital companion as underwriting similar forms of cognitive extension?

Moving beyond the simple case of question-answer systems, which (to some) may not feature the sort of dynamic reciprocal engagement that warrants treatment in cognitive terms (see Palermos 2014b), we can surely entertain the possibility of future forms of computational agent that help to shape the trajectory of our mnemonic and creative endeavours. Imagine, for example, a future Siri agent that participates in the kinds of collaborative recall and elaborative rehearsal processes mentioned above (Sutton et al. 2010; Smart 2010). In this case, despite the fact that we are interacting with a technological (as opposed to a biological) agent, there seems little reason to discount the Siri agent as an intrinsic part of some ‘socially-extended’ cognitive processing routine. In fact, once we consider the availability and accessibility of such synthetic agents, we may be inclined to see them as more apt for cognitive incorporation than their non-synthetic human counterparts.

## Human-Extended Machine Cognition

The agent that lies at the heart of an episode of extended cognizing is, in almost all cases, an individual human agent. In other words, it is the cognitive processes of the individual human agent that are deemed to be extended as a result of some form of bio-technological or bio-social bonding. Consistent with this view, many proponents of extended cognition have embraced what has come to be known as the Hypothesis of Organism-Centred Cognition (HOC) (Clark 2008). This is presented as the idea that

Human cognitive processing (sometimes) literally extends into the environment surrounding the organism. But the organism (and within the organism, the brain/CNS) remains the core and currently the most active element. Cognition is organism centered even when it is not organism bound. (Clark 2008, p. 139)

By situating a biological organism at the heart of an extended cognitive system, the HOC mandates a bio-centric view of extended cognition. That is to say, the HOC encourages us to see cognitive extension as something that is centered on a particular biological agent, typically a human individual.

In the current section, I want to challenge this idea and suggest that the Internet enables us to consider an entirely new form of extended cognitive organization. The kind of system I have in mind here is based on the idea that the points of contact we have with the online environment—the points at which we interface (and perhaps bond) with Internet-accessible resources—are able to function as bidirectional ‘plug points’. In other words, the rich array of devices that we now use to exploit the resources of the online realm are not just the points at which we plug into the online environment, they are also the points at which a variety of online systems are able to plug into us! It is these bidirectional points of contact with the Internet that enable us Internet-using human agents to be co-opted into extended nexuses of cognitively potent information processing, ones which are managed, monitored and maintained by various forms of machine-based intelligence. The result, I suggest, is that we can see the Internet, in combination with a multiplicity of artificial intelligence systems, cognitive computing platforms and machine learning algorithms, as providing the basis for forms of extended cognizing in which it is the *human agents* that form part of the physical machinery for non-biological forms of cognitive processing. For the sake of convenience, let us refer to this specific form of cognitive extension as Human-Extended Machine Cognition (HEMC).

The HEMC concept, it should be clear, alters our view as to the locus of cognitive control and agency within an extended cognitive organization. In addition to the idea that it is the cognitive routines of human individuals that are materially extended as a result of various forms of bio-technological and bio-social bonding (the conventional focus of active externalist theorizing), we now have to contend with the idea that it is the cognitive routines (and associated capabilities) of machine-based agents that are extended as a result of the sorts of (techno-social) bonding opportunities that are made available by the Internet. This idea is important, not only because it challenges the bio-centric bias of contemporary philosophical discourse, but also because it may help to advance the cause of artificial intelligence research. For if it is indeed our ability to enter into deep and complex relationships with extra-organismic resources that best explains the distinctive features of human intelligence (see Clark 2003), then perhaps the best way to extend the reach of extant artificial intelligence systems is to likewise focus on their ability to participate in materially hybrid forms of cognitive processing.

There will be much that is no doubt controversial here. The main point of contention for those seeking to defend the possibility of HEMC is likely to arise in respect of the notion of ‘machine cognition’ (e.g. does it make sense to talk of machines as cognitive systems?). In addition, for those who seek to argue that the Internet facilitates the emergence of HEMC systems, there are likely to be additional points of tension regarding the extent to which human agents are apt for cognitive incorporation (do human agents, for example, exhibit the requisite kind of functional poise that would enable them to be considered as constitutive elements in extended cognitive circuits?).^14^

In respect of the first of these points—the one relating to machine cognition—I assume that many philosophers of a materialist persuasion will not wish to deny the possibility that some kind of non-biological system could be accorded cognitive status. In other words, they will not wish to rule out the possibility that some form of synthetic computational system could engage in processes that are seen to be cognitive in nature. The issue of machine cognition is thus one that is closely aligned with a number of foundational issues in cognitive science, including those associated with computationalism (Piccinini 2012) and computational functionalism (Polger 2009).

In respect of the second point—the one relating to the functional poise of the human social environment—I suggest that it helps to consider the degree of social penetration and entrenchment that has been established by the Internet. Over the past several decades, the Internet has become so much a part of our everyday activities that it is sometimes hard to distinguish where the Internet stops and society begins (see Smart and Shadbolt 2014). And relative to this level of social penetration, it is possible, I suggest, to think of the human social environment as a more-or-less ever-present resource that can be harnessed, exploited and (to some extent) manipulated in more-or-less the same sort of manner as an individual human agent might interact with an array of readily available physical artefacts. I suggest, therefore, that the Internet enables us to regard the elements of the human social environment (from the HEMC perspective) in pretty much the same sort of way as we regard the elements of the physical environment from the perspective of individual (organism-centered) forms of extended cognition.

The HEMC concept is clearly deserving of a more detailed treatment than the one that can be offered here. In spite of the cursory coverage, however, the implications of the idea relative to ongoing debates concerning (e.g.) organism-centered cognition and the transformative potential of bio-technological hybridization should be relatively clear. The possibility of HEMC thus strikes at the heart of the HOC, forcing us to question the bio-centric intuitions upon which it is based. In addition, the HEMC concept helps to broaden the scope of current philosophical debates. It enables us, for example, to establish a useful point of contact between issues of complementarity in both the extended mind literature (see Sutton 2010) and the machine intelligence literature (e.g. Kapoor et al. 2008, Branson et al. 2014). There is, moreover, a growing sense that the Internet enables us to tap into human capabilities for the purposes of accomplishing tasks that were previously beyond the reach of our species (Michelucci and Dickinson 2016; Hendler and Berners-Lee 2010). One has only to look at the burgeoning literature on human computation systems (Michelucci 2013; Law and von Ahn 2011) and citizen science systems (Lintott and Reed 2013; Cardamone et al. 2009) to appreciate the growing interest in issues of socio-computational and bio-technological hybridization. It is here, I suggest, that we can begin to see how the notion of HEMC helps to bring a range of important inter-disciplinary linkages into sharper focus. In particular, the interest in materially heterogeneous cognitive organizations in contemporary philosophy of mind is perfectly aligned with the current efflorescence of research efforts (spread across a range of scientific disciplines) that seek to integrate human agents into episodes of machine-based computational processing (see Michelucci 2013;2016).

## Conclusion

The application of active externalist theorizing to the Internet gives rise to the notion of Internet-extended cognition—the idea that the Internet can serve as part of the physical fabric that realizes human mental states and processes. This is, in fact, a concept that warrants further philosophical and cognitive scientific attention. Firstly, the Internet is a major part of the technological and informational environment in which human agents are materially embedded. To an ever-greater extent such technologies are, in the words of Floridi (2014), ‘reshaping human reality’. For this reason, the Internet is an important target of active externalist theorizing. This is especially so if the philosophical debate is to keep pace with the changing profile of human cognitive and epistemic endeavours.

A second reason why the concept of Internet-extended cognition is important is because of the way that it helps to reveal issues that are not as readily apparent in cases involving technologically low-grade resources (e.g. conventional pen and paper resources). Such issues can, on occasion, strike at the heart of philosophical debates concerning the extended mind. We saw, for example, that a consideration of Internet-extended cognition highlights the need for a subtle re-crafting of the criteria that are sometimes used to authenticate cases of cognitive extension (see Section 2.2). We also saw that a consideration of Internet-extended cognition can introduce us to relatively novel forms of cognitive extension. These include conjoined minds (see Section 4), Internet-enabled forms of socially extended cognition (see Section 6), and (perhaps most importantly) forms of cognitive extension in which it is the human social environment that serves as the extended supervenience base for the cognitive routines of machine-based systems (see Section 7). What is important to bear in mind here is that none of these points of potential philosophical progress are revealed by the sort of (technologically low-grade) cases of cognitive extension that tend to be focus of the majority of extant philosophical treatments. This is one of the reasons why Internet-extended cognition is of crucial importance to the philosophy of mind and cognitive science communities.

Throughout the current paper, I have sought to survey a range of issues that arise in relation to the notion of Internet-extended cognition. One of these issues concerns the extent to which the Internet is able to support real-world cases of cognitive extension. This applies as much to the case of technologically extended cognition (see Section 2 and 3) as it does to the case of socially extended cognition (see Section 6). One of the important points to emerge from the discussion of this issue was the way in which our contact with the Internet is changing as a result of shifts in the technological landscape (e.g. the proliferation of wearable technologies) and shifts in the representation of online information (e.g. the move towards a Web of Data). This highlights a crucial issue when it comes to discussions about the potential of the Internet to support the emergence of extended cognitive systems: the Internet is in a state of rapid technological flux and we should not therefore assume that the range of opportunities for cognitively potent forms of bio-technological hybridization are necessarily exhausted by a selective focus on one particular form of interactive engagement with the online world.

Another issue concerned the putative transformation of our cognitive and epistemic capabilities as a result of Internet-based forms of cognitive extension (see Section 5). In general, the Internet has been seen to betoken a significant expansion in our epistemic capabilities (Ludwig 2015; Bjerring and Pedersen 2014); however, I have attempted to sound a note of caution here. While we might assume that Internet-extended cognition leads to an effective supersizing of our epistemic power and potential, things are not so straightforward when the epistemic status of the Internet-extended cognizer is evaluated with respect to virtue-theoretic epistemological theories, especially those that emphasize the role of cognitive ability in securing claims of positive epistemic standing (Greco 2007; 2010; 2012).

All of the issues surveyed in this review are ones that highlight the value and importance of inter-disciplinary collaboration. Given the nature of the target technological system (i.e. the Internet), it is likely that collaboration between the philosophy of mind, the cognitive science and the WAIS community will be of particular importance in shaping the course of future philosophical debates. Such forms of inter-disciplinary collaboration are likely to be especially relevant when it comes to our understanding of the cognitive and epistemic impacts of the Internet, both for ourselves and the machines we build. Given that the Internet is likely to play an increasingly important role in shaping the profile of our social, cognitive and epistemic endeavours, it is imperative that we ensure that the properties of emerging technologies are ones that will enable future forms of intelligence (both human and machine) to thrive and flourish in the new cognitive ecology of the online, digital world.

## Footnotes

1 For the purposes of this paper, I will treat these terms as largely synonymous. The notion of an ‘Internet-extended mind’ should thus be seen as equivalent to the notion of an ‘Internet-extended cognitive system’.

2 A range of criteria have been discussed in the extended mind literature. Aside from the criteria of trust, availability and accessibility (Clark 2010b), other criteria include the need for continuous forms of reciprocal interaction between the agent and bio-external artefact (Palermos 2014b) and the need for the external resource to be subject to what is called ‘epistemic possession’ (Clowes 2015). Some have even sought to develop multi-dimensional frameworks to support the assessment and evaluation of extended cognitive systems (see Heersmink 2015).

3 Something similar is, in fact, suggested by Clark and Chalmers (1998). When it comes to our understanding of what it is that makes something a cognitive process, for example, Clark (2010b) notes that ‘The argument by Clark and Chalmers assumes that we do possess some such understanding, and that it is rooted, roughly speaking, in our implicit knowledge of the distinctive functional role of cognitive processes in guiding intelligent behavior’ (p. 55).

4 The nature of the bio-external resource (e.g. its technological sophistication) actually makes very little difference here. To help us see this, we can reflect on two extended mind scenarios. In the first scenario, an agent interacts with a smartphone in order to access information that is stored locally on the device (i.e. on the smartphone’s hard disk). In the second scenario, the same agent interacts with the same smartphone in order to access information that is stored remotely (i.e. on the Internet). From the perspective of the availability criterion, the first scenario is roughly equivalent to the classic Otto case: if the smartphone is to count as part of the supervenience base for Otto’s dispositional beliefs, then it had better be the case that the smartphone is available to Otto as and when he needs it. The same, of course, is also true in the second scenario; however, in this case, something extra is required. Thus, in addition to the mere availability of the smartphone, we also need to be sure that the smartphone is properly connected to the Internet. If this is not the case, then the fact that the smartphone is available will count for nothing.

5 Consider, for example, how the same Wikipedia article might be displayed on a smartwatch versus a conventional desktop computer.

6 The envisaged capabilities, in this case, are somewhat similar to those of existing smartphone apps, such as Leafsnap (Kumar et al. 2012) and ApLeaf (Zhao et al. 2015).

7 See Giere (2012, p. 201) for similar views about the inadequacy of information transfer in the case of distributed cognition.

8 When it comes to issues of collective cognitive processing, for example, the structure of the communication network that mediates the flow of information between individual problem-solving agents is often deemed to be of crucial explanatory relevance in accounting for particular performance outcomes (e.g. Kearns 2012).

9 This lack of attention is unfortunate, since the topic of conjoined minds gives rise to a number of important issues. It seems, for example, that the idea of shared supervenience bases for mental states and processes provides a form of inter-individual cognitive contact that is just as profound and important as that evoked by recent work on brain-to-brain interfaces (Pais-Vieira et al. 2015; Grau et al. 2014).

10
^10^ Consider, for example, the number of people that might rely on a common body of online information, such as that provided by Wikipedia.

11
^11^ Of course, what is deemed to be significant, and thus worthy of discussion, is itself something that is, at least in part, determined by the social environment, e.g. the beliefs, attitudes, values and interests of the social group.

12
^12^ Such a shift in philosophical focus is likely to be desirable, in any case, especially when one considers the impact of online social networking systems (e.g. Facebook, Twitter, Instagram, etc.) on the structure of human social processes. There is a potential danger here of the philosophical debate failing to keep track of the rapidly shifting reality of human social behavior.

13
^13^ There are, in fact, multiple examples of such programs already available. See, for example, ‘Bird Song Id’ by Isoperla (http://us.isoperlaapps.com/BirdSongIdUSA.html).

14
^14^ The nature of these tensions, it should be clear, echo those that are encountered in the mainstream extended mind literature (Adams and Aizawa 2010; Clark 2010a).
